# A Rare Cause of Acquired Factor X Deficiency in an 87-Year-Old Female

**DOI:** 10.1155/2021/1138329

**Published:** 2021-11-17

**Authors:** Ayrton Bangolo, Trupti Waykole, Bilal Niazi, Chandini Sajja, Mahabuba Akhter, Bhavna Gupta, Sameh Elias

**Affiliations:** ^1^Department of Internal Medicine, Hackensack Meridian Health Palisades Medical Center, 7600 River Road, North Bergen, NJ 07047, USA; ^2^Department of Hematology and Oncology, Hackensack Meridian Health Palisades Medical Center, 7600 River Road, North Bergen, NJ 07047, USA

## Abstract

Factor X deficiency is a rare coagulopathy that can be inherited or acquired. Acquired factor X deficiency has been associated with plasma cell dyscrasias, amyloids, and use of vitamin K antagonists. Of plasma cell dyscrasias, most cases in the literature have been associated with multiple myeloma with or without concomitant AL amyloidosis. Here, we present a rare case of acquired isolated factor X deficiency in an elderly patient with immunoglobulin A (Ig A) monoclonal gammopathy of undetermined significance (MGUS). Herein, we highlight a rare cause of acquired factor X deficiency, and we hope to contribute to the growing literature of plasma cell dyscrasias associated with factor X deficiency.

## 1. Introduction

Factor X is one of the vitamin K-dependent serine proteases. It plays a crucial role in the coagulation cascade, as the first enzyme in the common pathway of thrombus formation [1]. In the presence of factor Va, calcium, and phospholipid membrane, factor X forms the prothrombinase complex that accelerates thrombin formation [2]. Acquired cases of factor X deficiency can be seen in patients with plasma cell dyscrasias as well as amyloidosis [3]. Patients with factor X values less than 10 percent can have a high risk of spontaneous major bleeding, and those with more than 40 percent are usually asymptomatic [4]. The most common bleeding symptom reported at all levels of severity of the deficiency is epistaxis [2]. The inherited form affects one person in every one million [5, 6]. Here, we present an asymptomatic 87-year-old female with monoclonal gammopathy of undetermined significance (MGUS) who was incidentally found to have isolated factor X deficiency during a preoperative evaluation. With this case report, we hope to contribute to the growing literature of plasma cell dyscrasias associated with factor X deficiency.

## 2. Case Presentation

This case is about an 87-year-old female with a past medical history of thyroid cancer, primary hyperparathyroidism, and monoclonal gammopathy of undetermined significance (MGUS). The patient was referred to the hematology office by the primary care physician for evaluation of prolonged prothrombin time (PT) and partial thromboplastin time (PTT) during surgical preoperative evaluation. Notably, the patient was diagnosed with papillary thyroid cancer two months prior to presentation and was scheduled to undergo a thyroidectomy the month following our office encounter. She was diagnosed with immunoglobulin A (IgA) MGUS 3 years prior to presentation during evaluation of hypercalcemia (workup for multiple myeloma was done at the time, but the patient was found to have primary hyperparathyroidism). An immunofixation 6 months prior revealed a lower level of IgA with kappa light chain, consistent with stable MGUS. She denies any hematuria, epistaxis, postmenopausal vaginal bleeding, or easy bruising. The patient had prior surgeries without preoperative prolongation of PT and PTT or excessive intraoperative bleeding.

On physical examination, she was found to be hemodynamically stable without any signs of bleeding. Laboratory workup revealed a prolonged PT, PTT, and an isolated decrease in the activity of factor X which corrected upon mixing studies as seen in Table 1. A diagnosis of factor X deficiency was made, and no therapy was indicated given the patient was asymptomatic at the time of diagnosis.

The patient received 25 international units per kilogram (IU/Kg) of factor X concentrate on the day of thyroidectomy and the next 2 days following the surgery without any major bleeding complications.

## 3. Discussion

Acquired factor X deficiency is a rare coagulopathy that can be seen in patients with plasma cell dyscrasias as well as amyloidosis [3, 7]. Coagulopathy, with clinically relevant bleeding events, is not an unusual phenomenon for patients with systemic amyloidosis secondary to multiple myeloma (MM) [7]. However, clinically relevant bleeding in patients with symptomatic MM, without associated amyloidosis, has been recently reported in the literature [7]. Monoclonal gammopathy of undetermined significance (MGUS) cells are characterized by the same primary genetic mutations as in myeloma and represent a precursor condition with an average 1%/year risk of progression into myeloma or other lymphoproliferative disorders [8]. Some cases of essential monoclonal gammopathy can cause acquired coagulopathy as the immunoglobulin can interact with coagulation factors [9]. To our knowledge, this will be the first case report of acquired factor X deficiency associated with MGUS without concomitant MM or AL amyloidosis.

Factor X deficiency is associated with bleeding in individuals who have a factor X activity of less than 10 percent. This includes excessive bleeding after invasive procedures and intracranial, umbilical cord, joint, and muscle bleeding [10]. The risk of bleeding correlates relatively well with factor X activity levels in plasma [11]. Our patient's factor X activity level was 15% and thus she did not have any bleeding symptoms. But, she was scheduled to receive prophylactic treatment before and after surgery.

Plasma-derived factor X concentrates are used for treating bleeding. Dosing is 25 international units/kg, repeated daily until bleeding stops [12]. In institutions where a factor X concentrate is not available, bleeding can be treated with a 3-factor or 4-factor prothrombin complex concentrate or fresh frozen plasma [12]. Single-factor replacement therapy is preferred over multifactor therapy as reported in the series of study by Peyvandi et al. [13]. The aim of treatment should be maintaining a factor X level that is at least 10% to 20% normal for minor bleeding. For more serious hemorrhage, a factor X level that is greater than 40% of normal should be the goal [2]. Our patient had a factor X activity of 15%, and she did not report any bleeding symptoms; thus, she did not need any therapy at the time of our encounter. However, she received prophylactic therapy with factor X concentrate before and after the thyroidectomy, given the invasive nature of the procedure, with good outcome.

## 4. Conclusion

Acquired factor X deficiency is a rare coagulopathy that has been associated with amyloidosis, secondary to multiple myeloma (MM). Only recently, cases of acquired factor X deficiency in MM patients, without concomitant amyloidosis, have been reported. Circulating immunoglobulins in plasma cell dyscrasias interact with coagulation factors and lead to the deficiency. Monoclonal gammopathy of undetermined significance (MGUS) has been associated with acquired coagulopathies by the same mechanism. However, to our knowledge, no case of factor X deficiency in the setting of MGUS has been previously reported in the literature. With this case report, we portray a rare, acquired cause of factor X deficiency and we aim to contribute to the growing literature of plasma cell dyscrasias-associated coagulopathies.

## Figures and Tables

**Table 1:** Coagulation factors, prothrombin time, and partial thromboplastin time activities before and upon mixing studies.

	Laboratory values	Laboratory values upon mixing
Prothrombin time	15.6 seconds (9.1–12)	10.2 seconds
Partial thromboplastin time	36 seconds (24–33)	28 seconds
International normalized ratio	1.5 (0.9–1.2)	
von Willebrand factor antigen	153% (50–200)	
von Willebrand factor activity	119% (50–200)	
Factor II activity	85% (50–154)	
Factor VII activity	110% (51–186)	
Factor VIII activity	115% (56–140)	
Factor IX activity	83% (60–177)	
Factor X activity	15% (76–183)	83%
Factor XI activity	83% (60–150)	
Factor XII activity	102% (50–150)	
Factor XIII activity	124%	

## Data Availability

All data generated or analyzed during this study are available from the corresponding author upon request.
